# Incidence of Gynecological Cancers Is Rising in Northern Iran: A Population‐Based, Serial Cross‐Sectional Joinpoint Regression Study

**DOI:** 10.1002/hsr2.73296

**Published:** 2026-09-30

**Authors:** Mohammad Amouzadeh‐Lichahi, Sahand Karimzadhagh, Nooshin Zaresharifi, Azadeh Jafari, Paridokht Karimian, Maryam Jafari, Gholamreza Sadeghi, Mohammad Ali Yazdanipour

**Affiliations:** ^1^ Student Research Committee, School of Medicine, Anzali International Campus Guilan University of Medical Sciences Rasht Iran; ^2^ Department of Internal Medicine, School of Medicine Shahid Beheshti University of Medical Sciences Tehran Iran; ^3^ Clinical Research Development Unit of Ghaem Hospital Guilan University of Medical Sciences Rasht Iran; ^4^ Neuroscience Research Center, School of Medicine Guilan University of Medical Sciences Rasht Iran; ^5^ Department of Pathology, School of Medicine Guilan university of medical sciences Rasht Iran; ^6^ Department of Obstetrics and Gynecology, Al‐Zahra Hospital Reproductive Health Research Center Rasht Iran; ^7^ Guilan Cancer Registry Guilan University of Medical Sciences Rasht Iran; ^8^ Neuroscience Research Center Poursina Hospital Guilan University of Medical Sciences Rasht Iran

**Keywords:** cancer, corpus uteri, gynecological cancer, ovary, trend

## Abstract

**Background and Aims:**

Gynecological cancers are a major global health burden, with ovarian, uterine, and cervical cancers being the most common types. Despite rising national incidence rates, recent comprehensive data for Gilan Province remain limited. This study investigated the incidence trends of gynecological cancers in Gilan Province, Northern Iran, from 2014 to 2018.

**Methods:**

A repeated cross‐sectional analysis was conducted using data from the National Cancer Registry and Guilan University of Medical Sciences. Newly diagnosed cases of gynecological cancers were identified and classified according to ICD‐O‐3. Statistical analyses included descriptive statistics, one‐way ANOVA for age group comparisons, and joinpoint regression to evaluate trends.

**Results:**

A total of 1058 cases were analyzed. The overall incidence rate rose by 98.2% over 5 years from 2014 to 2018, increasing from 10.72 to 21.25 cases per 100,000 individuals. The mean age of patients was 55.43 ± 3.63 years, showing no significant variation over time. Ovarian cancer was the most common type of gynecological cancer (42%), followed by corpus uteri (29.5%) and cervical cancer (15.2%), with 5‐year incidence rates of 35.15, 24.7, and 12.73 cases per 100,000 individuals, respectively. Joinpoint regression suggested significant annual increases in both crude (APC = 19.19%, *p* = 0.040) and age‐adjusted rates (APC = 22.22%, *p* < 0.001). While the cumulative 5‐year incidence of gynecological cancers was 83.78, significant geographic disparities were observed; Bandar‐e Anzali exhibited the highest rate (148.57), followed by Astaneh‐ye Ashrafiyeh (103.88), whereas Talesh reported the lowest (56.66).

**Conclusion:**

The incidence of gynecological cancers in Gilan province almost doubled during the study period, highlighting a substantial public health concern linked to long‐term burden and mortality rates. Interventions should prioritize community‐wide education, timely referrals, expanded access to diagnostic services, and enhanced awareness of early symptoms.

## 1 Introduction

Cancer is one of the leading causes of mortality worldwide, ranking second globally, only to cardiovascular diseases, and is the third in Iran, following accidents and cardiovascular conditions [1, 2]. Approximately one in five women will develop cancer in a lifetime, and around one in twelve will die from it. Alarmingly, the global cancer burden is projected to rise by 76% by 2050, with an estimated 35 million new cases annually, up from 20 million in 2022. Gynecological cancers collectively are a significant part of this burden, ranking as the fourth leading cause of cancer‐related death among women worldwide [3, 4, 5, 6]. Globally, cervical cancer is the most common gynecological malignancy and the fourth most frequent cancer among women in both incidence and mortality, with around 660,000 new cases and 350,000 deaths in 2022 alone, highlighting the substantial global burden of gynecological cancers. Following cervical cancer, which has a global incidence rate of 3.3%, other major gynecologic malignancies include uterine corpus cancer (4.3%), ovarian cancer (3.4%), vulvar cancer (0.5%), and vaginal cancer (0.2%) [4, 7].

In Iran, gynecological cancers, particularly those affecting the cervix, uterus, and ovaries, represent a growing public health concern, with uterine cancer ranking as the fifth most common malignancy among Iranian women [8, 9]. Between 1990 and 2016, studies indicated a significant increase in the incidence of reproductive tract cancers among women in Iran, rising from 2.5 to 12.3 cases per 100,000 women. The most prevalent female cancers in Iran are cervical, uterine, and ovarian cancers [9, 10, 11]. These cancers exhibit strong associations with age, not only in incidence but also in clinical outcomes and complications [12]. Globally, cervical cancer tends to occur at younger ages compared to other major malignancies such as breast, lung, and ovarian cancers [13]. An epidemiological study in Iran observed that the average age at diagnosis for ovarian cancer is increasing, while the average age for cervical cancer diagnosis has decreased. The overall national incidence and mortality rate of cervical cancers were reported at 2.14 and 0.93 per 100,000 women, respectively [14].

Disparities in gynecological cancer outcomes also exist across racial and geographic lines. In the United States, for instance, Black women face disproportionately higher mortality rates from reproductive tract cancers compared to other racial groups, possibly because of delays in diagnosis and unequal access to healthcare [15]. Early detection and timely intervention are critical for improving survival and reducing disease burden [16]. A key part of guiding public health policy is understanding the local epidemiology of these cancers, including their distribution by age, geography, and potential risk factors. Unlike other cancers, no comprehensive study has been conducted in recent years on the prevalence of gynecological cancers in Gilan Province. Consequently, this study aimed to determine the incidence of gynecological cancers among women in Gilan Province from 2014 to 2018.

## 2 Method

### 2.1 Study Design and Setting

This population‐based, repeated cross‐sectional study was conducted in Gilan Province, Northern Iran. It utilized data collected from the National Cancer Registry (NCR) of the Ministry of Health and Medical Education (MOHME) and the Noncommunicable Diseases Unit of Guilan University of Medical Sciences (GUMS). The NCR applies standard quality control procedures, including duplicate removal and diagnostic verification, to minimize such biases. The study spanned 5 years, from January 1, 2014, to December 31, 2018, and included all newly diagnosed cases of gynecological cancers during this period. Notably, we followed the Strengthening the Reporting of Observational Studies in Epidemiology (STROBE) guidelines when reporting the study. The completed STROBE checklist is provided in the Supporting Information S1 [17].

### 2.2 Participants and Variables

All women with primary gynecological cancers in Gilan Province during the study period, identified via International Classification of Diseases (ICD‐O‐3 topography codes) from cancer registry records, were included. As this was registry‐based, all eligible cases were included, and non‐participation was not applicable. The primary outcome variable was the incidence of gynecological cancers per 100,000 population, stratified by year, cancer site, and age group. Key variables included age, cancer site (topography), morphological type, and tumor grade. Notably, population denominator data were sourced from the same registry, which provided age‐specific counts of the female population used to calculate crude incidence rates.

### 2.3 Ethical Consideration

The study utilized retrospective, fully anonymized, and de‐identified secondary data aggregated across the whole province by the National Cancer Registry. Because individual contact with patients was neither feasible nor applicable, the requirement for individual informed consent was waived and approved by the Guilan University of Medical Science Ethics Committee on March 13, 2024, prior to data collection and subsequent analysis (IR.GUMS.REC.1402.617). These comprehensive records were then systematically reviewed and analyzed for this study.

### 2.4 Statistical Analysis

Statistical analysis was performed using IBM SPSS Version 21.0 software. Descriptive statistics were reported using frequencies, percentages, means, and standard deviations [18]. One‐way ANOVA was used to assess differences in mean age across years. Age‐standardized incidence rates (ASR) per 100,000 person‐years were calculated using the direct method with the WHO world standard population [19]. Age groups were categorized as 0–14, 15–39, 40–44, 45–49, 50–54, 55–59, 60–64, 65–69, 70–74, 75–79, and 80+ years.

Joinpoint regression analysis was performed using Joinpoint Regression Program version 5.4.0 (National Cancer Institute) [20]. The analysis was conducted on both crude and age‐standardized rates. Regarding model selection, the following parameters were applied: a maximum of one joinpoint was allowed, given the 5‐year study period (2014–2018), as at least two observations are required between a joinpoint and each endpoint. The grid search method was used to identify joinpoints, with a minimum of two observations from a joinpoint to the beginning and end of the series. The log‐linear model (ln(*y*) = *xb*) was employed to calculate the annual percent change (APC) under the assumption of uncorrelated errors. Heteroscedasticity was addressed using the standard error calculated from the data. The weighted Bayesian Information Criterion (BIC) was used for model selection. A *p* value ≤ 0.05 was considered statistically significant. Additionally, Datawrapper was used to create illustrations [21].

## 3 Results

A total of 1058 newly diagnosed cases of gynecological cancer were identified between 2014 and 2018. The cumulative 5‐year incidence of gynecological cancers in Gilan province was about 84 new cases per 100,000 women. The incidence rate suggested an increase of 98.2% over 5 years, from 2014 to 2018, rising from 10.72 to 21.25 cases per 100,000 individuals. The mean age of patients was 55.43 ± 3.63 years, with no significant change over the 5‐year period (*p* = 0.145). The most common topographic location was the ovary, accounting for 42.0% of all cases, followed by the corpus uteri (endometrium) at 29.5% and the cervix uteri at 15.2%. Among these, cervical cancer experienced the most significant increase, rising by over twofold at 158%, followed by ovarian cancer, which increased by 86.6%, and cancer of the corpus uteri at 76.7% (Table 1).

**Table 1:** The demographic and clinical characteristics of gynecological cancers from 2014 to 2018.

		Year					
		Total *n* (%)	2014 *n* (%)	2015 *n* (%)	2016 *n* (%)	2017 *n* (%)	2018 *n* (%)
Total number of new cases		1058	134	171	196	287	270
Incidence per 100,000 patients		83.78	10.72	13.62	15.51	22.68	21.25
Age (mean ± SD)		55.43 ± 3.63	53.28 ± 11.66	57.02 ± 13.62	54.84 ± 14.13	56.10 ± 14.28	55.20 ± 13.4
Topography^a^	Vulva	16 (1.26)	0 (0.0)	5 (0.4)	5 (0.4)	4 (0.31)	2 (0.15)
	Vagina	11 (0.85)	1 (0.08)	1 (0.08)	2 (0.15)	3 (0.23)	4 (0.31)
	Cervix uteri	161 (12.73)	19 (1.52)	26 (2.07)	26 (2.05)	40 (3.16)	50 (3.93)
	Corpus uteri	312 (24.7)	44 (3.52)	43 (3.42)	57 (4.51)	89 (7.03)	79 (6.22)
	Uterus, NOS	92 (7.27)	7 (0.56)	22 (1.75)	10 (0.79)	35 (2.76)	18 (1.41)
	Ovary	444 (35.15)	59 (4.72)	70 (5.57)	91 (7.20)	112 (8.85)	112 (8.81)
	Other and unspecified female genital organs	19 (1.49)	4 (0.32)	3 (0.23)	5 (0.4)	3 (0.23)	4 (0.31)
	Placenta	3 (0.24)	0 (0.0)	1 (0.08)	0 (0.0)	1 (0.08)	1 (0.08)
Grade^b^	1	140 (13.2)	21 (15.6)	22 (13)	23 (11.7)	37 (13.0)	37 (13.8)
	2	142 (13.4)	23 (17.1)	13 (7.6)	27 (13.7)	46 (16.0)	33 (12.2)
	3	187 (17.6)	25 (18.6)	23 (13.4)	39 (19.9)	48 (16.7)	52 (19.3)
	4	25 (2.3)	1 (0.7)	8 (4.6)	3 (1.5)	4 (1.4)	9 (3.3)
	9	564 (53.5)	64 (48.0)	105 (61.4)	104 (53.2)	152 (52.9)	139 (51.4)
Morphology	Neoplasms, NOS	272 (25.7)	27 (20.1)	63 (36.9)	49 (25.0)	83 (28.9)	50 (18.5)
	Epithelial neoplasms, NOS	52 (4.9)	5 (3.7)	12 (7.0)	6 (3.1)	8 (2.8)	21 (7.8)
	Squamous cell neoplasms	109 (10.3)	13 (9.7)	17 (10.0)	18 (9.2)	27 (9.4)	34 (12.6)
	Adenomas and adenocarcinomas	305 (28.9)	49 (36.6)	37 (21.6)	60 (30.6)	85 (29.6)	74 (27.4)
	Cystic, mucinous, and serous neoplasms	227 (21.5)	30 (22.4)	32 (18.7)	42 (21.4)	61 (21.3)	62 (23.0)
	Other morphology	93 (8.7)	10 (7.5)	10 (5.8)	21 (10.7)	23 (8.0)	29 (10.7)

^a^ The incidence of each gynecologic cancer type has been calculated as the number of new cases per 100,000 and is provided in parentheses. NOS: not otherwise specified.

^b^ Grade I, well differentiated, Grade II, moderately differentiated, Grade III, poorly differentiated,

Grade IV, undifferentiated, anaplastic, Grade IX, differentiation or cell type not determined, not stated, or not applicable.

The most frequently observed morphological types were adenomas and adenocarcinomas, accounting for 28.9% of all cases, followed by neoplasms not otherwise specified (NOS) at 25.7%, and cystic, mucinous, and serous neoplasms, collectively, at 21.5%. The distribution of morphological types varied over the years. Adenomas and adenocarcinomas were most common in 2014 (36.6%), while neoplasms NOS had the highest percentage in 2015 (36.9%) (Table 1). Detailed incidence distribution data of morphological categories for each cancer location are presented in Table 2. The distribution of final grades varied, with most cases falling under grade 9 (53.5%), followed by grade three cases (17.6%).

**Table 2:** Distribution of malignant morphologies by female genital tract site.

Topography	Major morphology groups	Total, *n* (%)
Vulva	Squamous cell carcinomas (incl. keratinizing, NOS, Bowen disease)	9 (56.25)
	Other carcinomas (basal cell, Paget, melanoma)	5 (31.3)
	Neoplasm malignant, NOS	2 (12.5)
	Total	16 (1.5)
Vagina	Squamous cell carcinomas	4 (44.4)
	Adenocarcinomas (including clear cell)	2 (22.2)
	Lymphoma	1 (11.1)
	Neoplasm malignant/carcinoma NOS	4 (22.2)
	Total	11 (1.03)
Cervix uteri	Squamous cell carcinomas (all types, including in situ and microinvasive)	94 (22.4)
	Adenocarcinomas (including clear cell and adenosquamous)	15 (17.6)
	Neoplasms malignant/carcinoma NOS	51 (60.0)
	Total	161 (15.21)
Corpus uteri	Endometrioid adenocarcinoma	185 (59.3)
	Other adenocarcinomas (serous, mucinous, clear cell, papillary)	65 (20.8)
	Sarcomas (leiomyosarcoma, stromal sarcoma, carcinosarcoma, Malignant Mullerian Mixed Tumor)	23 (7.4)
	Neoplasms malignant/carcinoma NOS	29 (9.3)
	Other rare histologies	6 (1.9)
	Total	312 (29.48)
Uterus, NOS	Neoplasm malignant, NOS	72 (78.3)
	Adenocarcinomas/serous/mixed	8 (8.7)
	Sarcomas/carcinosarcoma	8 (8.7)
	Other	4 (4.3)
	Total	92 (8.69)
Ovary	Serous carcinomas (cystadenocarcinoma, papillary serous)	175 (39.4)
	Endometrioid/clear cell/mucinous adenocarcinomas	54 (12.2)
	Other adenocarcinomas/carcinomas NOS	59 (13.3)
	Germ cell tumors (dysgerminoma, yolk sac, teratomas, mixed)	22 (5.0)
	Sex cord–stromal tumors (granulosa, Brenner, others)	3 (0.7)
	Sarcomas/MMMT/carcinosarcoma	6 (1.4)
	Neoplasm malignant/tumor cells, NOS	120 (27.0)
	Total	444 (41.96)
Other and unspecified genital organs	Fallopian tube carcinomas (serous, papillary, adenocarcinoma)	11 (57.9)
	Other adnexal/parametrium/overlapping sites	8 (42.1)
	Total	19 (1.79)
Placenta	Choriocarcinoma and malignant neoplasm	3 (100)
	Total	3 (0.28)

The 5‐year incidence rates across the cities of Gilan province reveal notable variations. Bandar‐e Anzali exhibits the highest rate of incidence at 148.57, with 101 newly diagnosed cancer patients. Astaneh‐ye Ashrafiyeh follows with a rate of 103.88. Rasht city, the capital of the province, despite having the highest number of newly diagnosed patients (406), had a cumulative incidence rate of 84.69. In contrast, Talesh city reported the lowest incidence rate at 56.66. These differing rates may suggest various underlying factors potentially influencing incidence rates across the region, although further investigation is needed to confirm these hypotheses (Figure 1).

**Figure 1:**
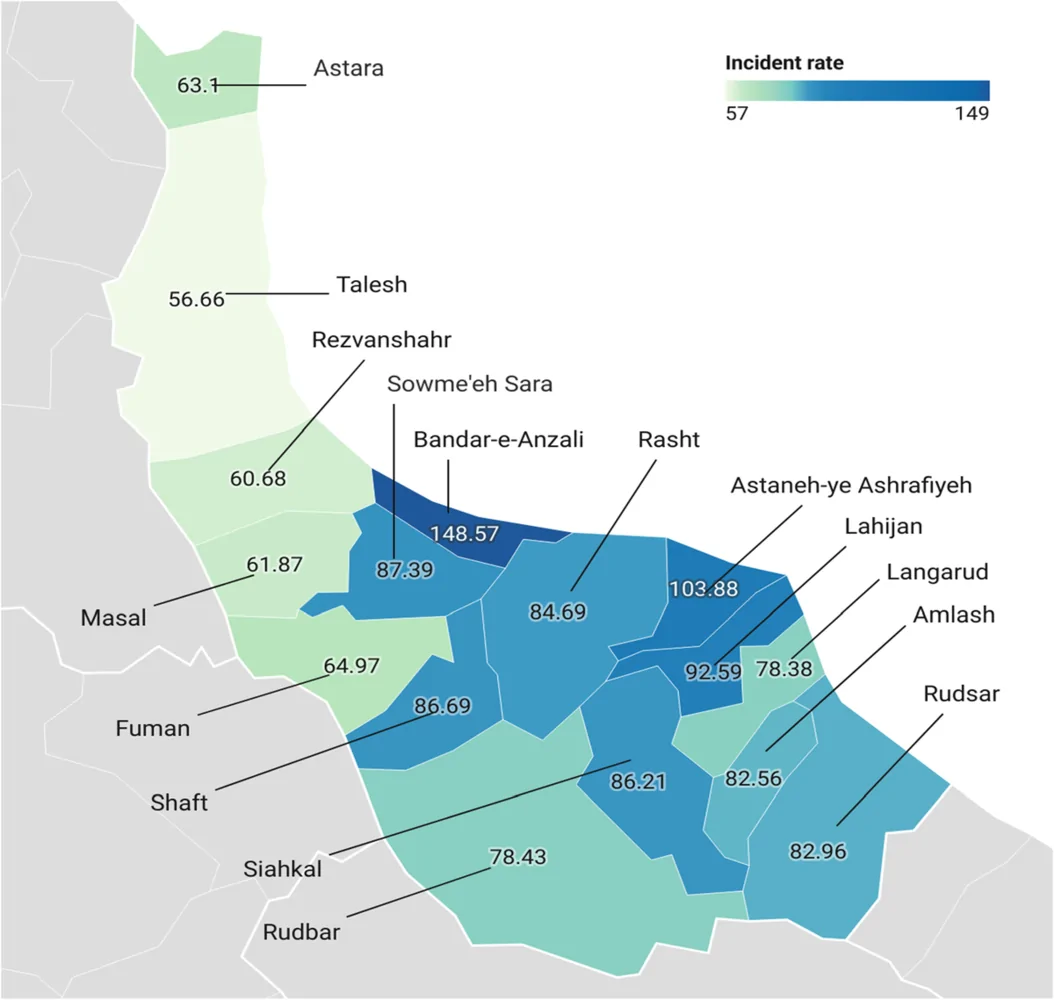
The total incidence rate of gynecological cancers across various cities in Gilan province from 2014 to 2018. The figure was illustrated with www.datawrapper.de.

In Gilan province, cancer incidence rates per 100,000 population show variable patterns across different cities and reproductive sites (Figure 2). Ovary cancer has the highest overall incidence, with Bandar‐e‐Anzali showing the highest rate at 54.43, followed by Siahkal (47.41) and Rudsar (42.17). Corpus uteri cancer incidence is also highest in Bandar‐e‐Anzali (42.66) and Astaneh‐ye Ashrafiyeh (36.52), while Rasht and Lahijan have moderate rates. Cervical cancer incidence is generally lower than ovarian and corpus uteri cancers, but is notably highest in Bandar‐e‐Anzali (25.01) and Siahkal (21.55), with lower rates in Astara (4.67) and Langarud (8.87). These findings highlight Bandar‐e Anzali as a high‐incidence area for all three major gynecological cancers. The observed variation among other cities may reflect regional differences in risk factors or diagnostic practices, requiring further investigations (Figures 1, 2).

**Figure 2:**
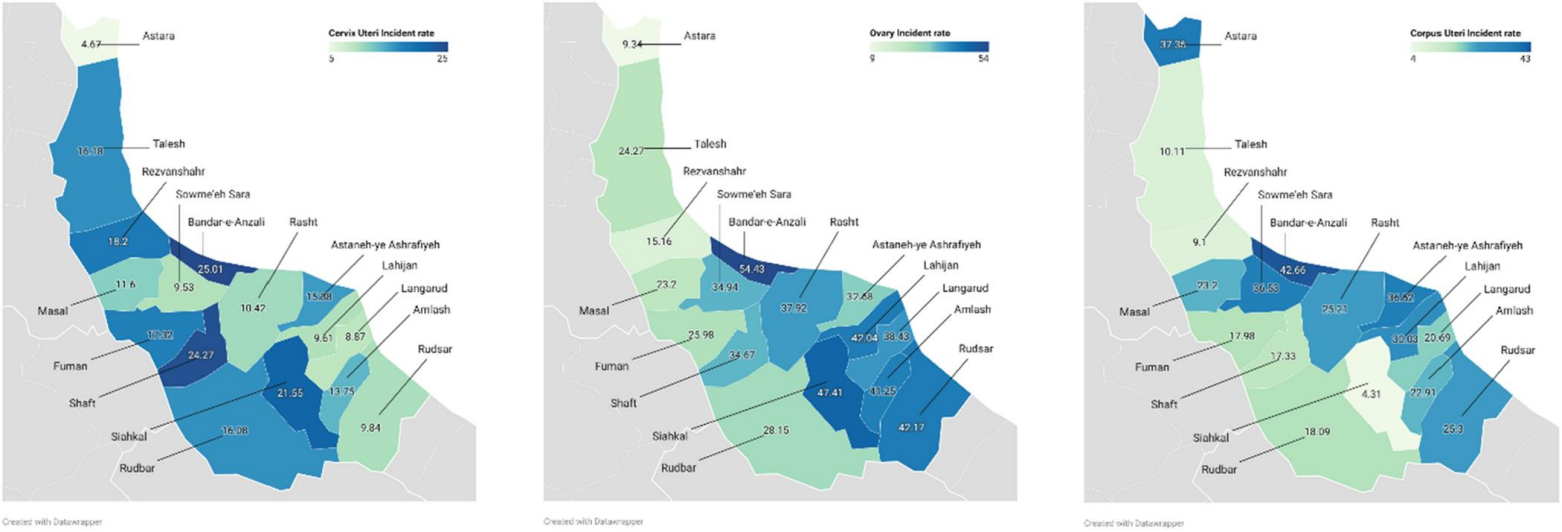
The overall incidence rates of the three most common gynecological cancers, Cervix uteri (left), Ovary (center), and Corpus uteri (right), across various cities in Gilan province from 2014 to 2018.

Joinpoint regression analysis of crude incidence trends from 2014 to 2018, suggested a significant overall annual percentage change (APC) of 19.19% (95% CI: 1.47–39.99; *p* = 0.040). The model identified a join point in 2016, dividing the trend into two distinct intervals: a sharp annual increase of 29.27% during the first segment (2014–2016), followed by a more gradual increase of 13.16% in the second segment (2016–2018). Similarly, the age‐adjusted incidence rate over the same period showed a highly significant overall APC of 22.22% (95% CI: 11.52–30.58; *p*< 0.001), confirming that the rising incidence trend persists after adjusting for age distribution (Figure 3, Table 3).

**Figure 3:**
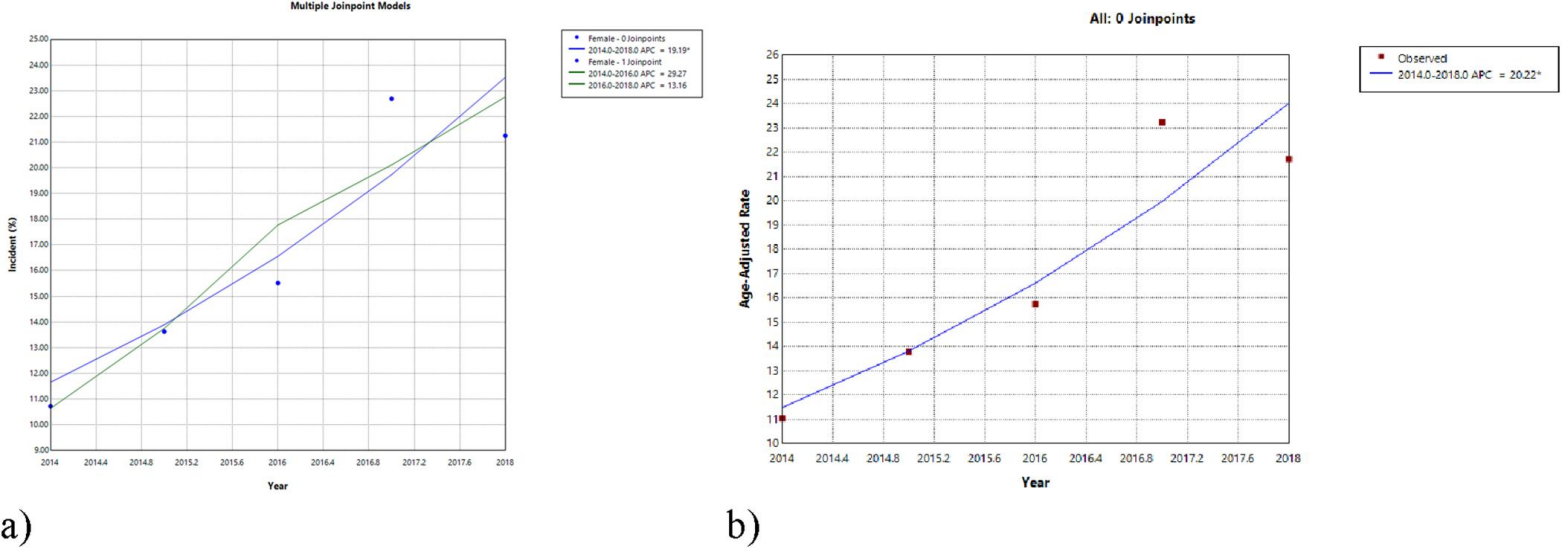
The annual percentage changes (APC) of incidents during 2014–2018, (a) crude, (b) age‐adjusted.

**Table 3:** The annual percentage changes (APC) of incidents during 2014–2018.

Cohort	Segment	Lower and upper endpoint	APC	95% CI	Prob > |*t*|
Female—0	1	2014–2018	19.1851^a^	(1.4708, 39.9917)	0.040310
Female—1	1	2014–2016	29.2705	—	—
	2	2016–2018	13.1620	—	—
Age‐adjusted rate		2014–2018	22.2231^a^	(11.5217–30.5791)	< 0.001

^a^ Indicated that the annual percentage change (APC) is significantly different from zero at alpha = 0.05.

## 4 Discussion

This population‐based analysis revealed a notable rise in the incidence of gynecological cancers in Gilan Province between 2014 and 2018, demonstrating a significant annual percentage change (APC) of 19.19%. Among the identified malignancies, ovarian cancer was the most prevalent, followed by corpus uteri and cervical cancers. The overall mean age at diagnosis was 55.43 years, consistent with prior national findings. These patterns are broadly in line with the increasing burden of gynecological cancers reported both within Iran and globally [4, 22, 23]. However, these findings should be interpreted with caution, as enhanced diagnostic access and optimized reporting infrastructure across Gilan Province during this timeframe might affect the trends.

The upward trend in incidence aligns with prior national studies. Shafiee et al. reported increasing trends in ovarian and uterine cancers over a 15‐year period in Iran, while cervical cancer showed a modest decline [23]. The observed trends in Gilan partly mirrored this national profile, particularly regarding the rise in ovarian and uterine cancers. In contrast, we observed the most significant increase in cervical cancer within our population. Furthermore, Azizikia et al., in a report from the 2017 national data, identified Gilan as one of the provinces with a disproportionately high incidence of cervical and uterine cancers, highlighting the regional significance of this public health issue [24].

Globally, similar increases have been reported in countries with both very high and low Human Development Index (HDI) tiers, albeit for different reasons. In very high‐HDI countries, expanded diagnostic access, lifestyle modifications, delayed childbirth, and aging populations could contribute to rising incidence, especially of uterine and ovarian cancers. Conversely, in low‐HDI countries, cervical cancer remains dominant due to inadequate HPV vaccination and limited screening programs [22, 25, 26, 27]. In our study, the dominance of ovarian cancer may also reflect regional factors such as older age distribution, high rates of obesity, and limited access to early detection services in Gilan province.

Geographic variation across Gilan's cities was also notable. Bandar‐e Anzali recorded the highest incidence rate, while Rasht had the highest number of cases but lower crude rates due to its larger population. While the exact reasons for these disparities remain unclear, we hypothesize that they could be related to differences in access to healthcare facilities, diagnostic practices, or awareness levels. However, these explanations need further assessment and should be interpreted with caution. Similar patterns have been observed in studies from Yazd and Isfahan provinces [28, 29]. Future analytical studies are needed to identify the actual determinants of this geographic variation. For instance, in Isfahan province, ovarian cancer had the highest incidence rates, and the mean age at which gynecological cancers were diagnosed was 57.8 years. In a study by Amiri and Lofti [28] conducted in Yazd province, half of the newly diagnosed cases were individuals over 50 years old, and most of these women received diagnoses at advanced stages. In our study, consistent with previous research, the average age at diagnosis was 55.43 ± 3.63, but we couldn't verify the staging differences due to limited available data. This is concerning, as older age and late‐stage diagnosis are linked to worse prognosis and limited treatment options. Studies from Romania and Nigeria similarly reported a high proportion of late‐stage presentations in gynecological cancers [30, 31], highlighting the need for enhanced early detection strategies globally.

A recent study reported a 20‐year incidence prediction of gynecological cancers in Iran, noting that we will see an 80% increase from 2020 to 2040, with the vulva and corpus uteri experiencing the most significant rise [32]. Despite a higher incidence over time being multifactorial, northern Iran, particularly Gilan province, may indicate a faster pace than other areas in the country.

While this study provides valuable epidemiological insights, several limitations should be acknowledged. First, the registry lacked data on individual‐level lifestyle and biological risk factors, such as HPV infection status, obesity, reproductive history, and family history of malignancy. Second, our explanations for the observed geographic variations across Gilan's municipalities remain descriptive and hypothetical, as we could not adjust for localized healthcare access or regional screening behaviors. Third, a substantial proportion of tumors were classified as neoplasms not otherwise specified (NOS) (25.7%) and grade 9/unknown differentiation (53.5%). This is a common challenge in regional registries and likely reflects unstandardized reporting templates across mixed private and public diagnostic hubs, small specimen sizes from core biopsies that limit definitive grading, or a lack of advanced immunohistochemical staining resources in smaller local laboratories. Clinically, this high proportion of unspecified data limits our ability to evaluate precise subtypes and disease severity. Epidemiologically, however, we confirmed that the proportion of NOS and Grade 9 cases remained highly stable on an annual basis throughout the study period, indicating that the sharp rising trends observed are not an artifact of shifting classification practices over time. Nevertheless, these trends should be interpreted with caution due to the relatively short 5‐year observation period, and longer‐term registry surveillance is required to confirm these trajectories definitively. To improve registry utility in future studies, we recommend strengthening regional pathology infrastructure through centralized review, implementing mandatory standardized reporting templates for gynecological cancers, and expanding institutional access to diagnostic subtyping panels. Finally, as with any large‐scale registry data, the potential for minor regional underreporting or misclassification cannot be entirely eliminated.

## 5 Conclusion

Our study revealed that the incidence of gynecological cancers among women in Gilan province almost doubled between 2014 and 2018, with ovarian, uterine, and cervical cancers being the most frequently diagnosed. This increasing trend highlights the urgent need for regionally adjusted healthcare planning and resource allocation. Public health interventions should prioritize community education, symptom awareness, and the establishment of timely diagnostic and referral pathways. Expanding access to specialized diagnostic services and addressing modifiable risk factors are critical steps toward managing the escalating burden of gynecological cancers in this region.

## Author Contributions

**Mohammad Amouzadeh‐Lichahi:** conceptualization, investigation, writing – original draft, validation, methodology, visualization, data curation, software, formal analysis. **Sahand Karimzadhagh:** writing – review and editing, writing – original draft. **Nooshin Zaresharifi:** conceptualization, investigation, validation, methodology, project administration, resources, supervision, data curation. **Azadeh Jafari:** investigation, validation, methodology. **Paridokht Karimian:** investigation, validation, methodology. **Maryam Jafari:** writing – original draft, data curation, investigation. **Gholamreza Sadeghi:** resources, data curation, validation. **Mohammad Ali Yazdanipour:** formal analysis, software, methodology, validation.

## Funding

The authors have nothing to report.

## Ethics Statement

The study received approval from the Guilan University of Medical Science Ethics Committee on March 13, 2024, prior to data collection and subsequent analysis (IR.GUMS.REC.1402.617).

## Conflicts of Interest

The authors declare no conflicts of interest.

## Transparency Statement

The corresponding author, Nooshin Zaresharifi, affirms that this manuscript is an honest, accurate, and transparent account of the study being reported; that no important aspects of the study have been omitted; and that any discrepancies from the study as planned (and, if relevant, registered) have been explained.

## Supporting information


Supporting File

## Data Availability

Due to confidentiality issues, the datasets generated and analyzed in the current study are not publicly available but can be obtained from the corresponding author upon request.
